# Supplementary material for: Postpartum women’s prospective acceptability of long-acting HIV prevention approaches in Kenya: a qualitative study
Source: BMC Health Serv Res. 2025 Aug 20;25:1109. doi: 10.1186/s12913-025-13286-4 (PMC12366025; doi:10.1186/s12913-025-13286-4)
Supplement: Supplementary file 1 — Supplementary Material 1. [file 12913_2025_13286_MOESM1_ESM.docx]

**Supplemental file 1**

**Participant IDI guide**

# LONG-ACTING PrEP

Now I would like to talk to you about new forms of PrEP that might be available soon. Currently, only daily oral PrEP is available for pregnant and postpartum women. Other types of PrEP may be available in the future. One option approved for pregnant and postpartum women is a flexible vaginal ring that you insert once a month to prevent HIV. Another option is an injection that is given by a healthcare provider every two months. This means that the medication stays in your body for two months. This form of PrEP is equally effective at preventing HIV as daily oral PrEP. Since this is a new type of drug, researchers are still studying if it is safe for pregnant women and their baby. So far researchers have found it to be safe, but there isn’t as much data on safety like we have for daily oral PrEP.

1. Do you think pregnant and postpartum women in general would prefer the daily pills, vaginal rings once a month, or the injections every couple of months to prevent HIV? Why?
2. If you were given the choice, would you prefer a long-acting injectable, vaginal ring, or daily oral form of PrEP? Why?
3. What information about long-acting injectable PrEP or a vaginal ring would pregnant and postpartum women need to know to feel comfortable taking it?
4. What concerns do you have about a long-acting injectable PrEP?
   1. Probe: Any other concerns?
   2. Probe (if not mentioned as a concern): Safety data
   3. Probe (if not mentioned as a concern): Two-month timeline
5. If these additional choices were offered to you, how would that have influenced your PrEP taking, or discontinuation?
   1. Offered after taking daily oral prep vs offered at the beginning?
   2. Would it be easier to adhere to a long-acting PrEP option compared to a daily oral PrEP?
6. Do you think this SMS support tool would be useful and helpful to have for a long-acting PrEP like the injection or vaginal ring?
   1. What messages would need to be added to support these other PrEP types?

# CONCLUSION

1. Before we end, is there anything else you would like to share with me?

***Thank you very much for your time and for all the helpful information you have provided. We will use this information you have provided in combination with information from others, to recommend solutions for better care of women and their children.***
